# Myosin-Powered Membrane Compartment Drives Cytoplasmic Streaming, Cell Expansion and Plant Development

**DOI:** 10.1371/journal.pone.0139331

**Published:** 2015-10-01

**Authors:** Valera V. Peremyslov, Rex A. Cole, John E. Fowler, Valerian V. Dolja

**Affiliations:** Department of Botany and Plant Pathology and Center for Genome Research and Biocomputing, Oregon State University, Corvallis, OR 97331, United States of America; Institut Jacque Monod, Centre National de la Recherche Scientifique, FRANCE

## Abstract

Using genetic approaches, particle image velocimetry and an inert tracer of cytoplasmic streaming, we have made a mechanistic connection between the motor proteins (myosins XI), cargo transported by these motors (distinct endomembrane compartment defined by membrane-anchored MyoB receptors) and the process of cytoplasmic streaming in plant cells. It is shown that the MyoB compartment in *Nicotiana benthamiana* is highly dynamic moving with the mean velocity of ~3 **μ**m/sec. In contrast, Golgi, mitochondria, peroxisomes, carrier vesicles and a cytosol flow tracer share distinct velocity profile with mean velocities of 0.6–1.5 **μ**m/sec. Dominant negative inhibition of the myosins XI or MyoB receptors using overexpression of the *N*. *benthamiana* myosin cargo-binding domain or MyoB myosin-binding domain, respectively, resulted in velocity reduction for not only the MyoB compartment, but also each of the tested organelles, vesicles and cytoplasmic streaming. Furthermore, the extents of this reduction were similar for each of these compartments suggesting that MyoB compartment plays primary role in cytosol dynamics. Using gene knockout analysis in *Arabidopsis thaliana*, it is demonstrated that inactivation of MyoB1-4 results in reduced velocity of mitochondria implying slower cytoplasmic streaming. It is also shown that myosins XI and MyoB receptors genetically interact to contribute to cell expansion, plant growth, morphogenesis and proper onset of flowering. These results support a model according to which myosin-dependent, MyoB receptor-mediated transport of a specialized membrane compartment that is conserved in all land plants drives cytoplasmic streaming that carries organelles and vesicles and facilitates cell growth and plant development.

## Introduction

How molecules and large macromolecular complexes are properly distributed and positioned is one of the central problems of cell biology. In relatively small prokaryotic cells, which lack endomembrane compartments, diffusion modulated by the cell’s metabolic status is a major mechanism at work for trafficking of both small molecules and large complexes [1]. Not so in much larger eukaryotic cells, where targeted transport of organelles and carrier vesicles, ER dynamics and cytosolic intermixing involve cytoskeletal transport networks encompassing microtubules and actin microfilaments with associated molecular motors [2–4]. Transport can be achieved by at least two mechanisms: active transport by receptor-mediated attachment of cargo to molecular motors and passive transport with cytosol flow or cytoplasmic streaming. It stands to reason that to establish streaming, a directional flow of motor-associated cargoes is required [5–7], thus making passive transport dependent on motor-driven active transport.

There are several well-studied eukaryotic models of active transport, including bud-directed traffic of membrane-bound organelles and vesicles in dividing yeast cells [8–11], long- and short-distance traffic in neurons, and melanosome transport to the cell periphery in melanocytes [3,4,12,13]. By and large, the mechanisms whereby these membrane compartments move are similar in that the motor proteins attach to cytoskeletal filaments on one end and to cargo-specific receptors on the other. In contrast, the mechanisms underlying cytoplasmic streaming are much less understood. The motor proteins driving streaming are known for *Drosophila* oocytes [6,14], *Caenorhabditis elegans* embryos [15] and rapidly moving fish keratocytes [16], whereas the motor cargoes that entrain cytosol are yet to be identified. It has also been proposed that the Arp2/3-dependent streaming in mouse oocytes is caused by the flow of actin filaments [17], although the origin of force driving the movement is not clear.

Plant cells present an ultimate case of perpetual and rapid cytoplasmic streaming, which reaches maximal velocities of up to 4 **μ**m/sec in *Arabidopsis* [18] and over 50 **μ**m/sec in a filamentous alga *Chara corallina* [19], compared to 0.4 **μ**m/sec in *Drosophila* oocytes [14]. Numerous studies over decades have converged on the actomyosin system as the driving force for the streaming [20,21]. More recently, genetic approaches demonstrated that myosin XI-K is the principal driver of organelle trafficking in plant cells, with myosins XI-2, XI-1 and XI-I providing redundant contributions [22–27]. Moreover, simultaneous inactivation of these myosins in a quadruple knockout mutant abolishes the process of endomembrane trafficking and, therefore, the cytoplasmic streaming as well [28]. Unexpectedly, subsequent work revealed that the principal cargoes of myosin XI-K and other myosins are not organelles as was broadly assumed, but rather a distinct endomembrane compartment defined by the membrane-anchored myosin receptors of the MyoB family that is universally conserved in all land plants [29,30]. This finding implied that the MyoB-associated compartment could be a main driver of cytoplasmic streaming. In addition, progressive elimination of myosins by combining multiple mutations results in a concomitant reduction in cell growth and plant stature [28] making myosins XI critical determinants of cell and organism size, and suggesting that cytoplasmic streaming is required for cell growth. Remarkably, a recent study demonstrates that acceleration of streaming by expression of a more rapidly moving myosin increases plant growth [18], further emphasizing the role of myosin-dependent streaming as a determinant of plant size.

Thus, despite significant progress in understanding cytoplasmic streaming, in no model system has a mechanistic connection been firmly established between a particular molecular motor, its specific cargo that entrains the cytosol, and the role of the resulting streaming in the cell’s biology [5]. It is also not clear if the saltatory trafficking of Golgi and other organelles depends on direct myosin binding or on transient entrance into localized streams of cytosol [22,31–33]. Here we show that the MyoB membrane compartment constitutes the myosin cargo that drives cytoplasmic streaming, which appears to transports organelles and carrier vesicles. We further demonstrate that the functional cooperation between myosins and their cognate MyoB receptors is required for cell expansion, as well as for plant growth and morphogenesis, thus defining the mechanism of cytoplasmic streaming and its role in plant biology.

## Materials and Methods

### Dominant Negative Inhibition of Organelle Trafficking

Genes encoding the *N*. *benthamiana* orthologs of Arabidopsis MyoB1 and MyoB2 were identified by searching the NCBI whole genome database. These genes were dubbed *NbMyoB1* and *NbMyoB2* respectively, and their 3’-terminal fragments encoding DUF593 and the rest of the protein up to the C-terminus, were PCR amplified and cloned into a binary vector pMDC32 under control of the 35S promoter. Triple haemagglutinin (3xHA) tags were added before the stop codons to facilitate detection of the recombinant proteins. A complete **β**-glucuronidase (GUS) ORF was also cloned and tagged with the 3xHA. All the resulting constructs were mobilized into *A*. *tumefaciens* GV3101 by electropopration. A plasmid expressing a 3xHA-tagged coiled coil/ globular tail region of *N*. *benthamiana* myosin XI-K was described earlier [22]. Full-length genomic copies of the *NbMyoB1* or *NbMyoB2* including ~2 kbp of upstream sequences harboring their putative promoters were PCR amplified and cloned. The GFP ORF was inserted upstream from the stop codons into the pMDC32 using *Sbf* I and *Pac* I sites as described for the Arabidopsis MyoB1 and MyoB2 [30]. The fluorophore-tagged markers for Golgi, mitochondria, peroxisomes or VAMP_721_ vesicles were also described previously [28,30]. A cDNA encoding **μ**NS of the mammalian reovirus [34] was a gift from Dr. M. Nibert. It was PCR amplified and inserted downstream from the mCherry or GFP ORF into pMDC32 plasmid using *Asc*I and *Pac*I sites.

The *N*. *benthamiana* leaves were co-infiltrated with the two Agrobacterium strains carrying an organelle-specific marker and a dominant-negative construct, both at OD_600_ = 0.5. The organelles were imaged 2 days post infiltration. The leaf fragments were immersed in water and observed using a Zeiss LSM510 confocal microscope equipped with a Plan-Apochromat 63x1.4 numerical aperture lens and a digital zoom 3.2X for larger objects such as Golgi stacks, or zoom 4.0X for smaller objects such as MyoB or muNS particles. The confocal images were acquired consecutively for 20 frames with no time delay between the frames and assembled into a single movie. In order to measure the speed of organelles, the speed for each individual object was calculated by dividing the distance travelled by the time the object was observed. The reported speed represents the average of all the measurements for all the organelles present at any time on all the recorded movies. All the organelles regardless of their motility that were present within given movies were indiscriminately tracked and their velocities measured and included in the calculation of average velocity as described above using video sequences of 20 consecutive frames. Movies were analyzed by ImageJ software (National Institutes of Health, Bethesda, MD) using the MTrackJ plugin. Only the objects that could be tracked on three or more (up to 20) consecutive frames were included in analysis. At least 100 individual objects for each organelle were manually tracked and their mean speed was calculated and analyzed using Microsoft Excel software.

### Immunoblot Analysis and Pull-Down Assays

The immunoblot analyses to detect proteins tagged either with the 3xHA or GFP were done as described [30] using rabbit polyclonal, HA- or GFP-specific antibodies, respectively (Rockland). The pull-down assays to investigate interactions of the *N*. *benthamiana* globular tail domains of myosin XI-K with its cognate receptor MyoB1 and the inclusions formed by GFP-**μ**NS (this marker was used instead of mCherry-**μ**NS because of higher sensitivity of the GFP-specific antibodies) were also conducted as previously described [30].

### Gene Knockout Lines

All knockout lines were homozygous for the specific mutant alleles and in the *A*. *thaliana* Columbia-0 background. The two double myosin knockout lines, for inactivated genes *xi-k xi-1* and *xi-k xi-2* [25], as well as the triple MyoB knockout *myob1 myob2 myob3* [30], were generated earlier and their null phenotypes were confirmed by RT-PCR. The quintuple synthetic knockout lines *xi-k xi-1 myob1 myob2 myob3* and *xi-k xi-2 myob1 myob2 myob3* were obtained by crossing corresponding double and triple gene knockout homozygous lines and genotyping the progeny by PCR.

### Cell Size Measurements

Plants were grown on a 10/14-hrs light/dark cycle at 24°C. Diameters of leaf abaxial mesophyll cells and lengths of leaf midrib epidermal cells were measured after the detached, fully developed leaves were fixed in ethanol and stained with 0.01% Toluidine blue [28]. The measurements of root hair length were described previously [28].

### Leaf, Stem and Root Growth Phenotypes and Flowering Time Analysis

The 30 plants of each line were used for measurements of plant height and leaf rosette diameter at 6 weeks post germination. Leaf rosette diameter was determined as the greatest distance between the apices of two opposite leaves [28]. Measurements of the primary root growth rate and root waviness are described in the S4 Fig legend.

For the flowering time analysis, plants were grown in growth chambers at 24°C, 10/14-h light/dark cycle after stratification for 3 days at 4°C. Flowering time for each individual plant was scored in days from sowing the seeds in soil to the day that the petals of first flower were visible. The flowering time for the each experimental line was defined as a mean time at which all plants formed the flowers.

### Statistical Methods

A 95% confidence interval (p<0.05) was considered to be statistically significant throughout this study. Statistical analysis of the organelle velocities has been done using the non-parametric Mann-Whitney U test because the distributions of the organelle velocities are skewed, do not fit the normal curve and do not pass the Anderson-Darling test for normality. The analyses of cell sizes were initially performed using directed pairwise t-test comparisons of each experimental variant to each respective control. Because some data sets did not pass Anderson-Darling test, these analyses were repeated using Mann-Whitney U test. Both of these approaches validated high statistical significance (p<0.001) of the difference between each of the two synthetic quintuple mutants and each of the respective three control data sets.

In the analysis of plant height and leaf rosette diameter, most of the data sets did not pass the normality test and therefore Mann-Whitney U test was used to statistically evaluate the data and to confirm highly significant (p<0.001) difference between each of the two synthetic quintuple mutants and respective controls. This approach has been also validated by application of one-way ANOVA followed by post-hoc Tukey HSD test for selected data sets. The Mann-Whitney test has also been performed for the growth to flowering transition. The sizes of root hairs and primary root growth rates were performed using a 1-sided t-test.

## Results

### The MyoB Compartment Contributes to Cytoplasmic Streaming and Organelle Trafficking

Identification of the distinct endomembrane compartment defined by MyoB receptors as the major myosin cargo [30] raised the question whether this compartment contributes to myosin-dependent organelle trafficking [22,27,28]. The Arabidopsis genome harbors as many as 16 functionally redundant MyoB paralogs, thus hindering gene knockout analysis of MyoB functions. Because all MyoBs share the conserved, myosin-binding DUF593 domain, overexpression of this domain should interfere with myosin binding to MyoBs, analogously to dominant negative inhibition of myosin activity upon overexpression of the conserved myosin cargo-binding domain [22,27]. To take advantage of a facile transient gene expression system in *Nicotiana benthamiana*, we isolated genomic clones of the *N*. *benthamiana MyoB1* and *MyoB2* orthologs of Arabidopsis MyoB1 and MyoB2, tagged each of them by inserting the GFP ORF, and found that the resulting proteins were targeted to the motile compartments similar to the MyoB1 and MyoB2 compartments described in Arabidopsis (S1A Fig) [30]. These validated clones were used to generate overexpression plasmids encoding the DUF593 domains of *N*. *benthamiana* MyoB1 and MyoB2 tagged by a triple hemagglutinin epitope (DUF593-1:HA_3_ and DUF593-2:HA_3_, respectively; S1B Fig).

**Fig 1:**
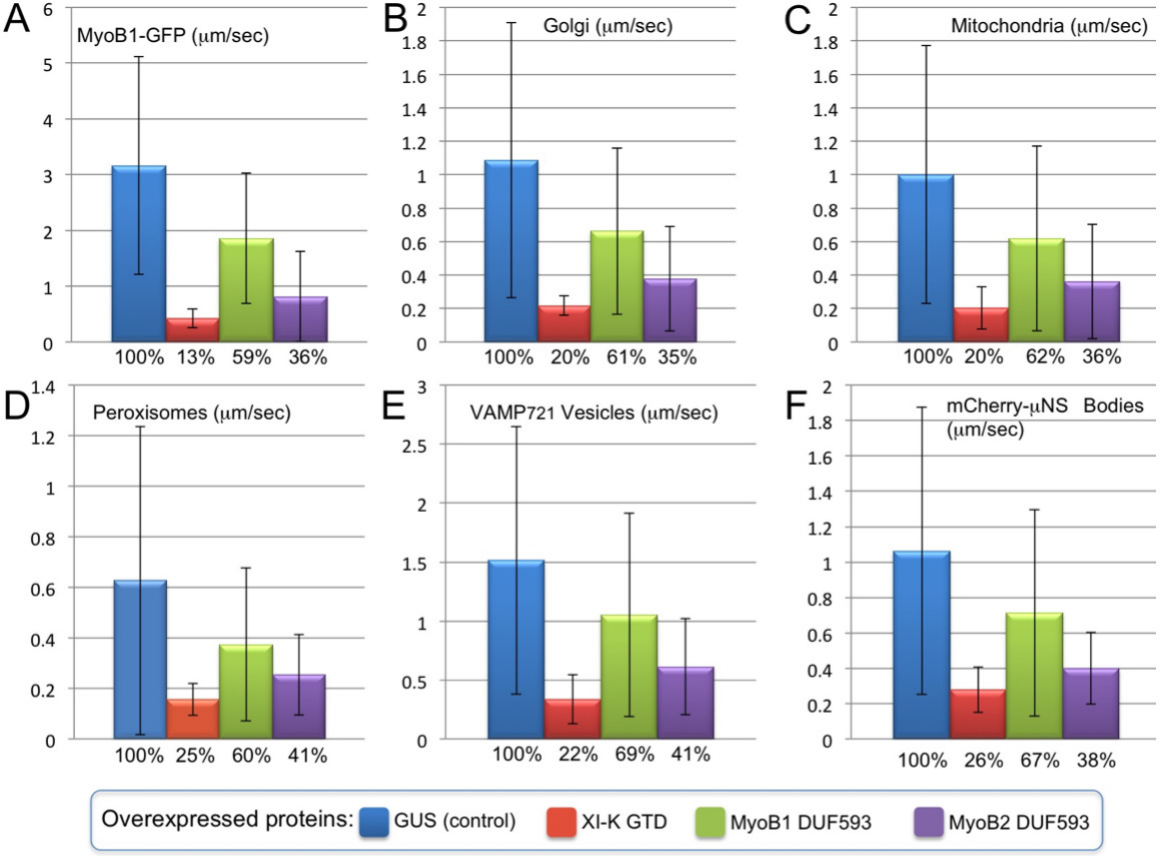
Dominant negative inhibition of particulate trafficking in *N*. *benthamiana* leaf epidermal cells upon transient overexpression of β-glucuronidase (GUS; control), the globular tail domain of *N*. *benthamiana* myosin XI-K (XI-K GTD) and the myosin-binding DUF593 domains of *N*. *benthamiana* MyoB1 and MyoB2 (MyoB1 DUF593 and MyoB2 DUF593). The mean velocities (**μ**m/sec) and standard deviations are shown for MyoB1 compartment tagged by full-size *N*. *benthamiana* MyoB1-GFP (A), Golgi stacks (B), mitochondria (C), peroxisomes (D), vesicles tagged by mCherry:VAMP_721_ (E) and inclusion bodies formed by the C-terminal fragment of mCherry-tagged, viral protein**μN**S, mCherry-**μ**NS (F). Percent of the mean velocity reduction relative to GUS control is shown below each column.

The overexpression of the cargo binding, globular tail domain of *N*. *benthamiana* myosin XI-K (GTD-K:HA_3_) was used as a positive control in all dominant negative inhibition experiments because this domain has a strong inhibitory effect on myosin-dependent transport [22]. The bacterial **β**-glucuronidase (GUS:HA_3_) was used as a negative control; mean velocity of organelle trafficking in cells expressing this protein was assigned 100% value in each experiment. In accord with a myosin-driven mechanism of MyoB compartment trafficking [30], GTD-K:HA_3_ expression significantly reduced the velocity of the endomembrane compartment tagged by MyoB1-GFP (Fig 1A; compare S1A and S1B Movie). Transient expression of the DUF593-1:HA_3_ or DUF593-2:HA_3_ also resulted in decreased velocities of MyoB1-GFP compartment trafficking, thus validating the dominant negative inhibition approach used in the following experiments (Fig 1A; S1C and S1D Movie). Given the similar expression levels of DUF593-1:HA_3_ and DUF593-2:HA_3_ (S1B Fig), the stronger inhibitory effect of the latter may be attributed to a relatively higher myosin-binding affinity of this domain.

To determine if the trafficking of myosin-MyoB compartment is mechanistically linked to trafficking of Golgi, we measured mean Golgi velocities upon overexpression of GTD-K:HA_3_, DUF593-1:HA_3_ and DUF593-2:HA_3_ relative to that in the presence of GUS:HA_3_ control. It was found that each of the three former protein domains exerted an inhibitory effect on the Golgi trafficking (Fig 1B; S2A–S2D Movie). Moreover, the extent of Golgi velocity reduction in each case was similar to that observed for the MyoB1-GFP compartment (cf. Fig 1A and 1B), although the mean velocity of the Golgi in a control variant was ~3-fold lower than that of MyoB1-GFP compartment (1.1 and 3.2 **μ**m/sec, respectively).

We have further performed dominant negative inhibition experiments for three additional types of organelles and carrier vesicles: mitochondria (Fig 1C), peroxisomes (Fig 1D) and the exocytic vesicle compartment defined by the v-SNARE VAMP_721_ (Fig 1E). The mean velocities of each of these compartments were not only affected by each of the three overexpressed protein domains, but the extents of velocity reduction were similar to those observed for either the MyoB1-GFP compartment or the Golgi (Fig 1A–1E). These results suggest that the myosin-MyoB compartment is a driver of cytoplasmic streaming, whereas the organelles follow in a wake of its movement (or reduced movement, when inhibited).

In previous studies of cytoplasmic streaming in plants including the most recent ones [18,35], streaming was evaluated by measuring velocities of either undefined membrane bodies or organelles. However, it is not known whether these streaming ‘reporters’ are directly attached to myosins and are the drivers rather than tracers of streaming. To address this problem, we assessed cytoplasmic streaming using foreign protein-based tracer particles. More specifically, we employed the non-structural protein **μ** (**μ**NS) of a mammalian reovirus incapable of infecting plants and thus acquiring plant myosin-binding receptors. Upon expression in cells including bacteria [1], this protein produces inclusions of various sizes via self-interaction of its C-terminal domain (amino acid residues 471–721) [34]. As expected, expression of this domain fused to mCherry (mCherry-**μ**NS) in *N*. *benthamiana* resulted in the formation of the numerous motile cytosolic inclusion bodies (S1C Fig; S3A Movie). Because the mCherry-**μ**NS fusion protein does not engage in any measureable interactions with myosin (S1D Fig), it does provide a bona fide inert tracer of cytoplasmic streaming. Among the broad size range of the tracer bodies, we selected those of 0.4–2.1 **μ**m (mean size of 0.99±0.35 **μ**m; n = 100). This size range corresponded to that of organelles and vesicles (vesicular clusters) measured in this study, although the surface and hydrodynamic properties of the tracer could have been distinct from those of endomembrane compartments.

The mean velocity of the mCherry-**μ**NS bodies was very similar to those of Golgi, mitochondria, peroxisomes or VAMP_721_ vesicles (Fig 1F). Furthermore, the extents to which the mCherry-**μ**NS velocity was reduced by overexpression of each GTD-K:HA_3_, DUF593-1:HA_3_ and DUF593-2:HA_3_ were very similar to those observed for Golgi, mitochondria, peroxisomes and VAMP_721_ vesicles (cf. Fig 1B–1F; S3A–S3D Movie). This result is compatible with a mechanism whereby the myosin-MyoB compartment actively drives cytoplasmic streaming which, in turn, carries passively floating cargoes including organelles, carrier vesicles and inert bodies alike.

**Fig 2:**
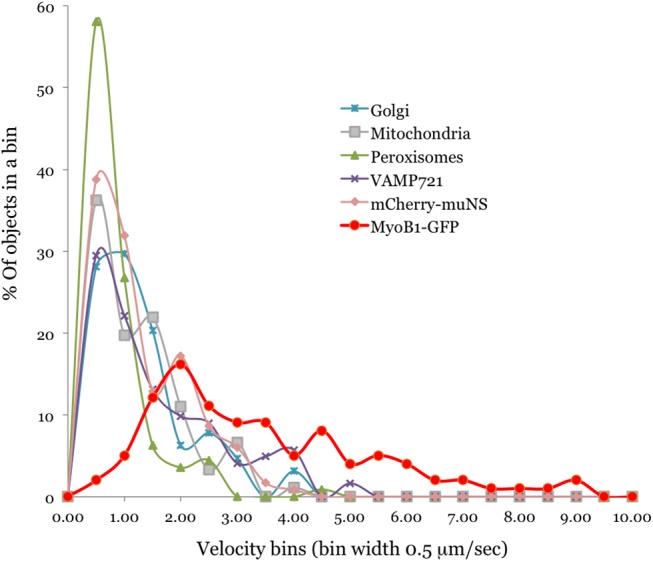
Velocity distribution profiles for organelles, VAMP_721_ vesicles, mCherry- μNS inclusion bodies, and MyoB1-GFP vesicle-like bodies in the presence of GUS (see legend to Fig 1). Distributions were plotted as % of objects exhibiting velocity range that falls in each bin of 0.5 **μ**m/sec width.

A comparative analysis of the velocity distribution profiles (Fig 2) was even more telling. The three organelles, VAMP_721_ vesicles and mCherry-**μ**NS bodies showed very similar motility patterns, with the majority of them (~80%) moving at ~0.5–1.5 **μ**m/sec or being nearly immobile. In contrast, only a minority (19%) of MyoB1-GFP compartment exhibited movement within this velocity range, whereas ~80% of MyoB1-GFP bodies moved at 2–9 **μ**m/sec (Fig 2). The differences between the motility pattern of the MyoB1-GFP compartment on one hand, and those of the organelles or inert bodies on the other, provide further support for distinct transport mechanisms involved. It seems reasonable to propose that the relatively low mean velocities of organelles, VAMP_721_ vesicles and inert particles are determined by the fraction of time they move with the localized cytosol flows versus staying within relatively immobile cytosol outside the flow routes.

To independently validate the role of MyoB receptors in cytoplasmic streaming supported by dominant negative inhibition approach, we employed gene knockout analysis and particle image velocimetry of mitochondria in *Arabidopsis* epidermal cells. This organelle was selected because its velocity profile is similar to those of other organelles and streaming tracer (Fig 2), and because of availability of facile vital staining. Velocity measurements of over 500 individual mitochondria in a Columbia control versus quadruple knockout mutant *myob1 myob2 myob3 myob4* demonstrated that inactivation of four MyoB receptors resulted in a statistically significant, ~24% reduction of the mean mitochondrial velocity (S2 Fig), providing direct genetic evidence of the MyoB function in organelle trafficking. Given that all 16 *Arabidopsis* MyoBs are expressed at the relatively low levels [30], it seems reasonable that elimination of the four MyoBs in a quadruple gene knockout reduces both the total pool of MyoBs and the velocity of cytoplasmic streaming roughly by a quarter. We were unable to measure the effect of MyoB elimination on the cytoplasmic streaming in *Arabidopsis* because the transgene expression level of the mCherry-**μ**NS tracer in this plant was insufficient to induce inclusion body formation.

Thus, both dominant negative inhibition experiments using the myosin-binding DUF593 domain of MyoB1 and MyoB2 in *N*. *benthamiana* and the limited gene knockout analysis using quadruple *myob1 myob2 myob3 myob4* mutant in *Arabidopsis* supported a direct role of the myosin-MyoB compartment in generating cytoplasmic streaming in plant cells.

### Myosins XI and MyoB Receptors Cooperate in Facilitating Cell Growth

Previous work provided genetic evidence of the functional significance of the MyoB1-3 transport compartments and their interaction with myosin XI-K in plant stem growth [30]. However, it was unclear if this compartment is a driver of the broader myosin functions in organ growth and cell expansion identified earlier [25,28,36], and if other myosins XI also execute their functions via cooperation with MyoB receptors. To answer these questions relevant to the role of the myosin-MyoB compartment in cytoplasmic streaming, we employed a synthetic gene knockout approach that targeted distinct combinations of myosin XI and *MyoB* genes. The term ‘synthetic’ is used throughout this study to emphasize that genes from two distinct gene families were simultaneously inactivated in a search for potential synthetic phenotypes versus incremental phenotypes of multiple knockout mutants of either myosin- or MyoB-encoding genes [28,30]. The myosins XI-K, XI-1 and XI-2 included in this analysis are highly expressed in vegetative plant and are implicated in cell expansion and plant growth [28]. Among these, myosins XI-K and XI-1 are closely related paralogs that belong to the same myosin XI subfamily, whereas myosin XI-2 is more distantly related paralog from distinct subfamily [37]. On the other hand, MyoB1-3 belong to the same subfamily I of the MyoB family [30]. The synthetic quintuple gene knockout mutants (s5KO), *xi-k xi-1 myob1 myob2 myob3* and *xi-k xi-2 myob1 myob2 myob3* were generated and characterized.

**Fig 3:**
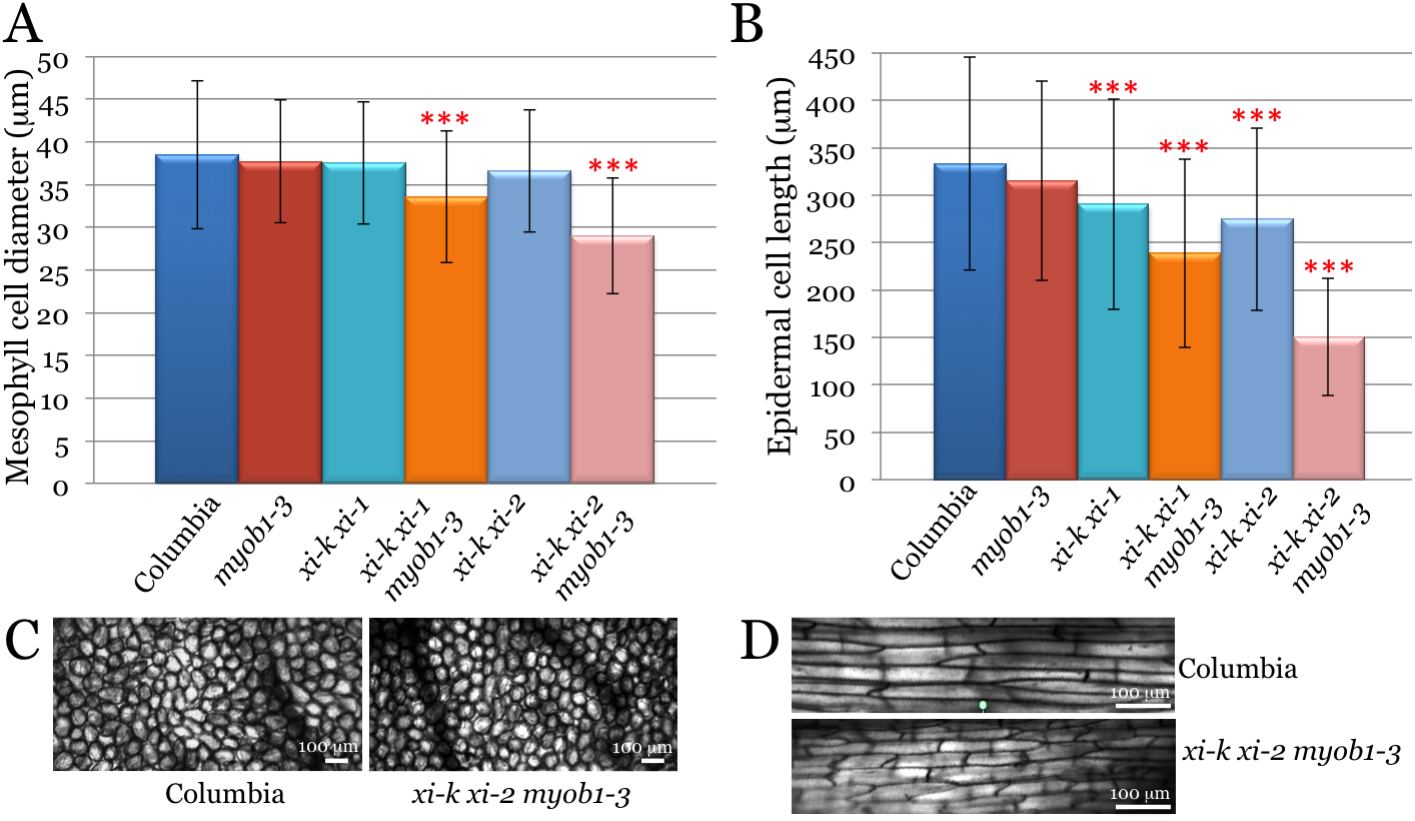
Size analysis of the leaf mesophyll and leaf midvein epidermal cells of the synthetic quintuple mutant Arabidopsis lines *xi-k xi-1 myob1-3* and *xi-k xi-2 myob1-3* compared to Columbia and parental lines *myob1-3*, *xi-k xi-1* and *xi-k xi-2*. Red asterisks indicate highly statistically significant differences (p<0.001) between each of the quintuple mutant line on one hand, and each of the corresponding control lines. (A) Mean diameters and standard deviations of the leaf mesophyll cells. (B) Mean lengths and standard deviations of the epidermal cells. (C and D) Representative images of the mesophyll and epidermal cells, respectively, of the Columbia and *xi-k xi-2 myob1-3* plant lines. Note apparently normal cell morphology in the quintuple mutant lines.

To investigate the potential contributions of the myosin-MyoB compartment to cell growth, we measured the sizes of three cell types (leaf mesophyll, leaf midrib epidermis, and root hairs) in the s5KO lines and compared those to the control lines Columbia-0, *myob1 myob2 myob3*, *xi-k xi-1* and *xi-k xi-2*. The mean mesophyll cell diameters in all these controls were not significantly different from each other (Fig 3A; p>0.05). On the other hand, this diameter in the *xi-k xi-1 myob1 myob2 myob3* s5KO was 13% smaller than that of Columbia (p<0.001 for pairwise comparisons with each of the three corresponding controls). The *xi-k xi-2 myob1 myob2 myob3* s5KO line exhibited an even stronger diameter reduction, by 25% relative to Columbia (Fig 3A; p<0.001 for all controls). The overall cell morphology was apparently normal in these s5KO mutants (Fig 3C).

As we have shown previously, the long epidermal cells at the lower surface of leaf midrib are sensitive indicators of myosins XI contributions to cell growth [25,28]. The analysis of the mean lengths of these cells revealed a moderate 5% reduction in the *myob1-3* 3KO line relative to Columbia; this reduction was not statistically significant at p>0.05 (Fig 3B). As expected, more significant length reductions of 13% and 17% were found for the *xi-k xi-1* and *xi-k xi-2* myosin double mutant lines, respectively (p<0.001 for pairwise comparisons with Columbia control). Strikingly, the epidermal cells of the s5KO lines exhibited much stronger length reduction effects of 28% and 55% in the *xi-k xi-1 myob1 myob2 myob3* and *xi-k xi-2 myob1 myob2 myob3* lines, respectively, compared to Columbia (Fig 3B; p<0.001). Their lengths were also significantly different from those of other two respective controls, double myosin knockout mutants and triple MyoB knockout mutans (p<0.001 for all four pairwise comparisons). However, the morphology of the midrib epidermal cells was unchanged even in the most severe s5KO mutant (Fig 3D).

An analogous investigation of fully expanded root hairs revealed a moderate, but statistically significant (p<0.001), 9% length reduction in a *myob1 myob2 myob3* knockout line relative to Columbia control (S3 Fig). On the other hand, there were no significant differences (p>0.05) between either *xi-k xi-1* versus *xi-k xi-1 myob1 myob2 myob3* lines, or *xi-k xi-2* versus *xi-k xi-2 myob1 myob2 myob3* lines. These results suggested that MyoB1-3 contribute only subtly to the polarized growth of root hairs, with the elimination of either of two myosin pairs having a dominant effect in this process.

Therefore, simultaneous inactivation of the three subfamily I MyoB receptors and two distinct sets of myosins XI demonstrated genetic interactions between these two types of proteins (at least in leaf cells) showing that, similar to myosin XI-K, myosins XI-1 and XI-2 also function in cooperation with MyoB receptors. Furthermore, the synergistic effects of cell size reduction in the s5KO mutants establishes the functional contribution of the myosin-MyoB compartment in the process of diffuse growth of the mesophyll and epidermal cells.

### Genetic Interactions between Myosins XI and MyoB Receptors Are Required for Stem and Leaf Growth in Arabidopsis

**Fig 4:**
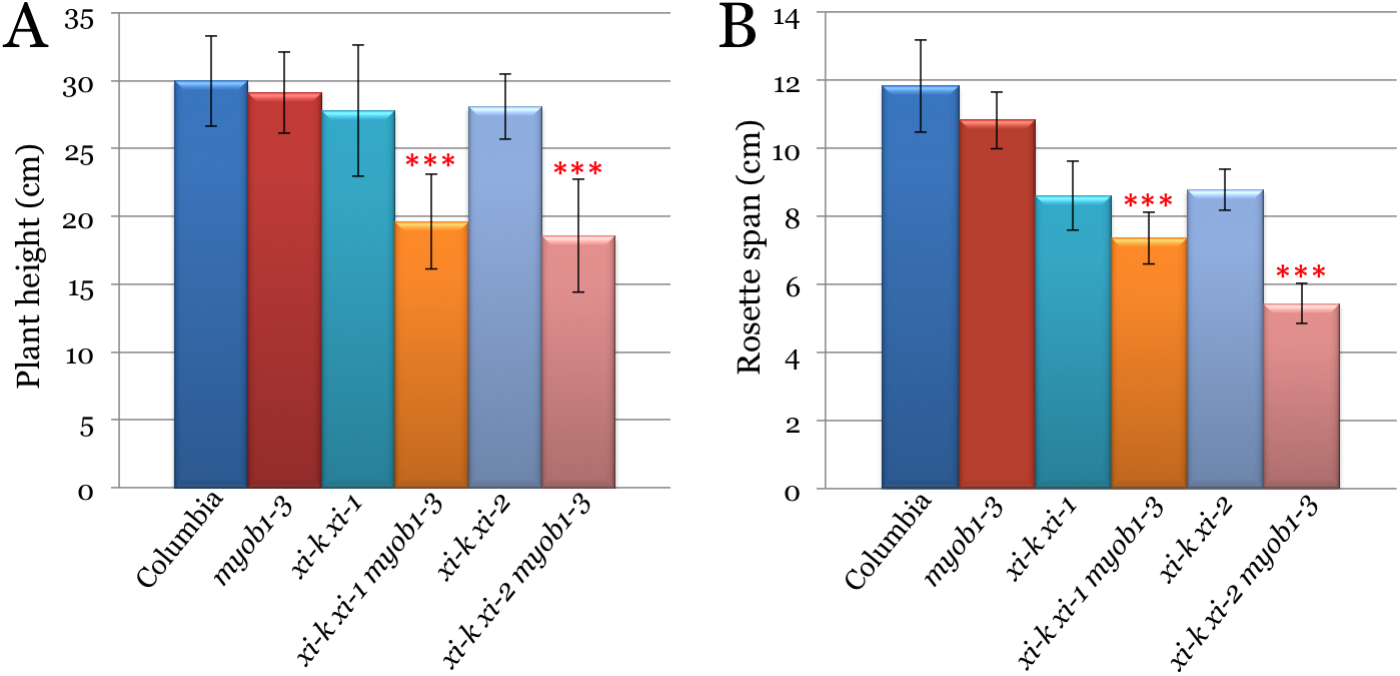
Growth phenotypes of the synthetic quintuple mutant Arabidopsis lines *xi-k xi-1 myob1-3* and *xi-k xi-2 myob1-3* compared to Columbia and parental lines *myob1-3*, *xi-k xi-1* and *xi-k xi-2*. Red asterisks, same as in Fig 3. (A) Mean plant heights and standard deviations. (B) Mean leaf rosette spans and standard deviations.

The growth phenotypes of the s5KO mutants were characterized under short day conditions (10/14 hrs light/dark) to favor vegetative growth over transition to flowering. The growth parameters of each s5KO line were measured and statistically evaluated against the three corresponding control plant lines. Analysis of the plant heights demonstrated that whereas *myob1 myob2 myob3* and *xi-k xi-1* lines exhibited only modest (~3% and ~10%, respectively) height reduction relative to the wild type Columbia line, the heights of the *xi-k xi-1 myob1 myob2 myob3* plants were reduced by 35% (Fig 4A). The differences between the mean plant height of the latter line compared to each of the three control lines were highly statistically significant (p<0.001). An analogous analysis of the *xi-k xi-2 myob1 myob2 myob3* s5KO line showed even more severe, 40% height reduction (Fig 4A; p<0.001 relative to each of the three control lines).

The leaf rosette size analysis of mutant lines showed similar, albeit an even stronger synergistic effect of simultaneous inactivation of the two myosin and three MyoB genes (Fig 4B). The leaf rosette spans of the *xi-k xi-1 myob1 myob2 myob3* and the *xi-k xi-2 myob1 myob2 myob3* plants were reduced by 48% and 54%, respectively. The statistical evaluation of the leaf rosette span differences between each s5KO mutant and each of the three corresponding controls were highly significant (p<0.001).

**Fig 5:**
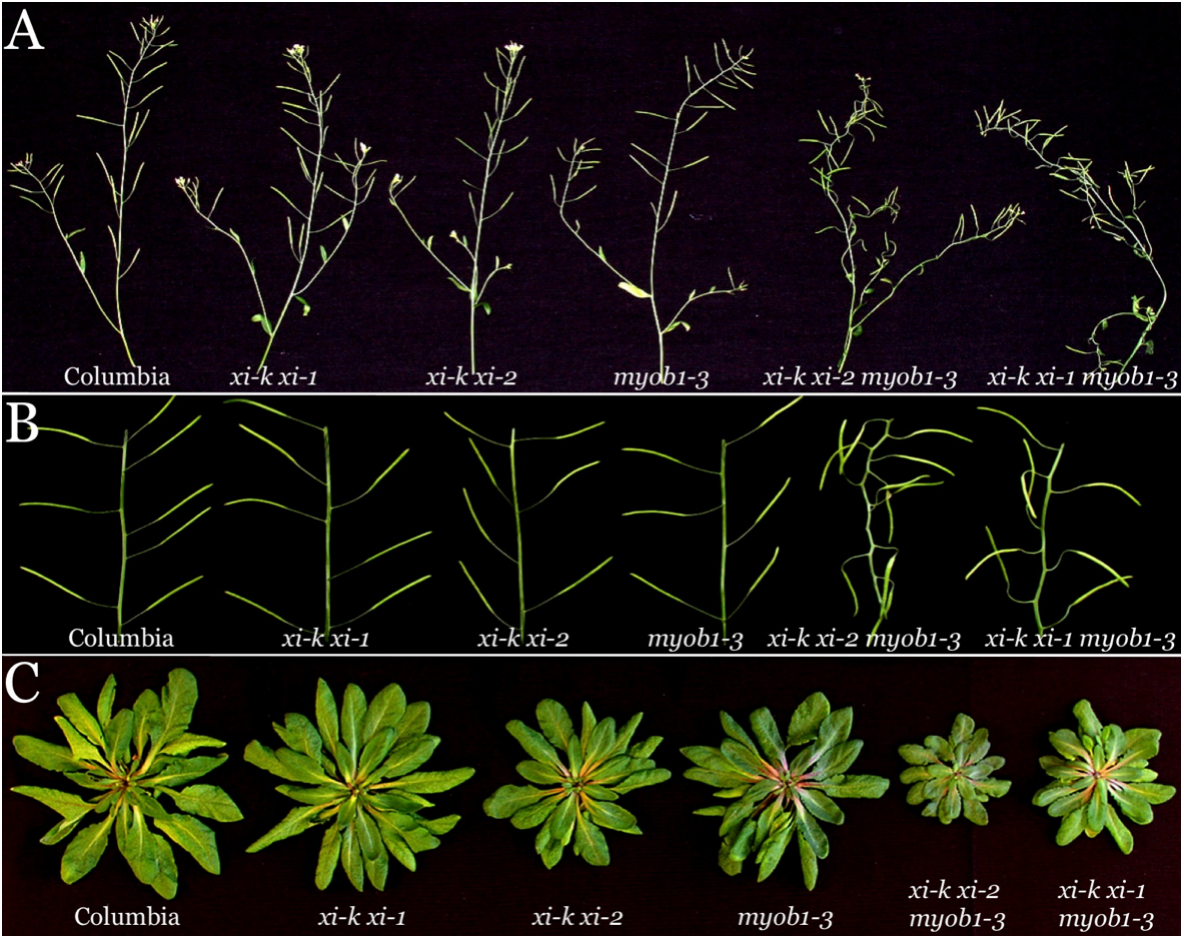
Representative images of the synthetic quintuple mutant Arabidopsis plants *xi-k xi-1 myob1-3* and *xi-k xi-2 myob1-3* compared to Columbia and parental lines *myob1-3*, *xi-k xi-1* and *xi-k xi-2* at 7 weeks after sawing. (A) The stems with siliques and flowers; note irregular stem shape in quintuple mutant plants. (B) Fragments of the stems with siliques; note irregular orientation of siliques relative to stems and misshaped silique stalks. (C) Leaf rosettes (stems and roots were cut off).

Interestingly, stem morphology was also affected in both s5KOs, but not in corresponding controls. As shown in Fig 5A, unlike control lines that exhibited relatively straight stems, the stems of both s5KO mutants were often bent and/or appeared wavy. Analogous morphological abnormalities were seen in the stalks connecting siliques to the stems. Whereas these stalks were straight and pointing slightly upward in four control lines, they were bent and pointing sharply upward or downward in both s5KOs (Fig 5B). In contrast, the overall rosette shape and leaf morphology in the s5KO mutants appeared normal despite the reduced rosette span (Fig 5C).

To investigate the role of genetic interactions between myosins and MyoB1-3 in root growth, the primary root growth rates were evaluated. These rates were not significantly different among Columbia, *myob1-3* 3KO and *xi-k xi-1* mutants (S4A Fig). The *xi-k xi-2* as well as the *xi-k xi-1 myob1 myob2 myob3* and *xi-k xi-2 myob1 myob2 myob3* mutant roots had only a very slight, but statistically significant, reduction in growth rate, compared to Columbia. The addition of the triple MyoB receptor knockout did not lead to an enhanced reduction in primary root growth rate in either of the s5KOs (S4A Fig) suggesting that, similar to root hair growth, root growth is regulated distinctly from stem and leaf growth, perhaps involving a different subset of myosins and MyoB receptors. On the other hand, simultaneous inactivation of two myosins and three MyoB receptors did affect root morphogenesis, resulting in the formation of wavy roots (S4B and S4C Fig), somewhat reminiscent of the stem bending phenotype apparent in the s5KO. Quantitative measurements of root waviness validated statistically significant differences between each s5KO mutant and corresponding controls (S4B Fig).

To determine if the phenotypic defects in synthetic myosin-MyoB mutants are indeed dependent on MyoB activity rather than incidental second-site mutations, we stably transformed the s4KO line *xi-k myob1 myob2 myob3* [30] with genomic clones of MyoB1 and MyoB2, each tagged by insertion of YFP ORF. Under short day conditions, the parental s4KO line exhibited defective stem growth and morphology compared to Columbia control (S5 Fig; note that bended stem defects are already apparent in s4KO). Conspicuously, both of these phenotypic defects were completely rescued by expression of MyoB1-YFP in the *xi-k myob1 myob2 myob3 MyoB1-YFP* line (S5 Fig). The MyoB2-YFP expression in the *xi-k myob1 myob2 myob3 MyoB2-YFP* line had a similar phenotypic rescue effect, although the stem growth was restored to 96% of that in the wild type, compared to 99.7% in the case of MyoB1-YFP (S5B Fig). In addition to validating the critical functions of the myosin-MyoB compartment in plant growth, these data demonstrated that MyoB1-YFP and MyoB2-YFP fusion proteins are functional, thus corroborating the biological relevance of their subcellular distribution patterns [30]. These data also implied that the functions of MyoB1 and MyoB2 in plant growth and morphogenesis are largely redundant.

Collectively, the presented results show that MyoB1-3 receptors interact genetically with myosins XI-K, XI-1 and XI-2 and that proper function of the myosin-MyoB compartment is required for normal growth of the stem and the leaf rosette. Furthermore, the distorted stem morphology and wavy root appearance caused by inactivation of the subsets of myosins and MyoBs clearly establish a morphogenic function of this compartment.

### Cumulative Effect of Myosins XI and MyoB Receptors on Flowering Time

**Fig 6:**
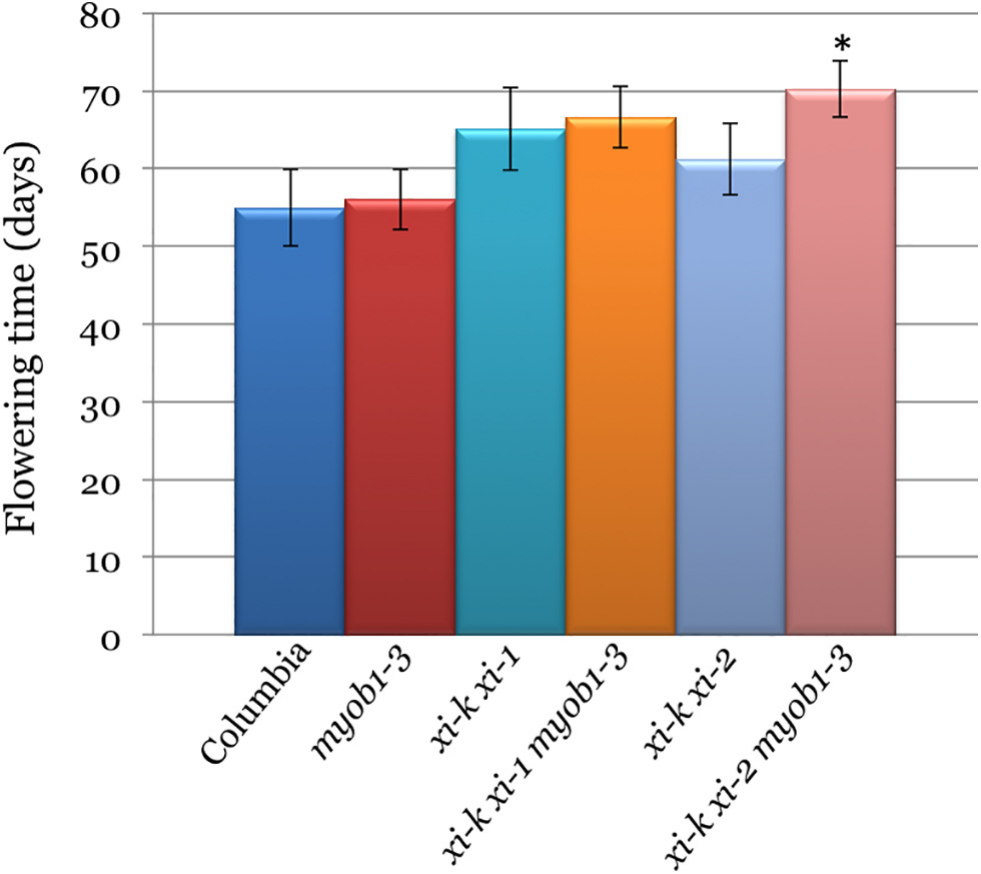
Mean flowering time and standard deviations for the synthetic quintuple mutant Arabidopsis lines *xi-k xi-1 myob1-3* and *xi-k xi-2 myob1-3* compared to Columbia and parental lines *myob1-3*, *xi-k xi-1* and *xi-k xi-2*. Black asterisk indicates marginally statistically significant difference (p = 0.05) between the *xi-k xi-2 myob1-3* and a corresponding *xi-k xi-2* control (the difference between this s5KO and Columbia or *myob1-3* controls was highly significant at p<0.001).

A significant decrease in the growth of the s5KO lines (Figs 4 and 5) prompted investigation of their developmental rate, of which onset of flowering is one of the key characteristics. As shown in Fig 6, inactivation of MyoB1-3 did not significantly affect flowering time compared to Columbia (p>0.05). On the other hand, inactivation of the myosins XI-K and XI-1 or XI-K and XI-2 did delay the flowering onset by 10 and 6 days, respectively, relative to Columbia (Fig 6; p<0.001 for *xi-k xi-1* and p<0.01 for *xi-k xi-2*). The simultaneous inactivation of the three MyoB receptors and either of the myosin XI pairs further exacerbated this delay. The most significant effect was observed for the *xi-k xi-2 myob1 myob2 myob3* s5KO line that flowered 15 days later than Columbia, 14 days later than *myob1-3* 3KO, and 9 days later than *xi-k xi-2* 2KO controls (Fig 4). It should be noted, however, that although the difference in flowering time between this s5KO and Columbia or MyoB 3KO controls was very significant (p<0.001), the statistical analysis pointed to only marginal significance of the difference with myosin 2KO (p = 0.05).

Thus, even though elimination of the MyoB1-3 receptors in itself does not delay flower development, it contributes to further flowering delay when combined with the double myosin XI knockouts. These results imply that the myosins and their membrane receptors influence flowering transition, a critical aspect of plant development and reproduction cycle.

## Discussion

Although cytoplasmic streaming is ubiquitous in eukaryotes, its exact mechanism and functions have not been determined in any of the model organisms, from plants where it was discovered nearly 250 years ago [5,21] to fungi [38] to animals [6]. Investigation of this process was confounded by the use of cytoplasmic streaming tracers that could themselves be cargoes transported by motor proteins, and thus were potential drivers of streaming, rather than a passive load that moves with the cytosolic flow. To solve this conundrum, we used the readily observable tracer mCherry-**μ**NS [34], which inherently lacks an ability to bind plant molecular motors. To determine the mechanism of cytoplasmic streaming, we combined the genetic approaches of inhibiting motor-driven transport with particle image velocimetry of the Golgi, mitochondria, peroxisomes, carrier vesicles, **μ**NS-mCherry bodies, and a membrane compartment defined by the MyoB receptors for myosins XI [30].

One significant finding of this study is identification of the two main modes of the vigorous endomembrane trafficking in plant cells. The first mode is a fast, incessant traffic of the principal myosin cargo, the MyoB-decorated membrane compartment that moves at a mean velocity of ~3 **μ**m/sec (Fig 1A). The second mode is represented by organelles, such as Golgi, mitochondria and peroxisomes (Fig 1B–1D) and carrier vesicles marked by the v-SNARE, VAMP_721_ (Fig 1E). The mean velocities of these four membrane compartments were similar at ~1 **μ**m/sec, with the large fraction of particles being nearly immotile. Intriguingly, the **μ**NS-mCherry inclusion bodies followed the latter mode, implying that organelles and carrier vesicles are also transported passively, with the cytosolic flow (Fig 1F). This interpretation is further supported by velocity distribution profiles peaking at mere 0.5 **μ**m/sec for organelles and mCherry-**μ**NS particles, in contrast to 2 **μ**m/sec for the MyoB compartment, a substantial fraction of which reaches velocities in excess of 7 **μ**m/sec (Fig 2).

**Fig 7:**
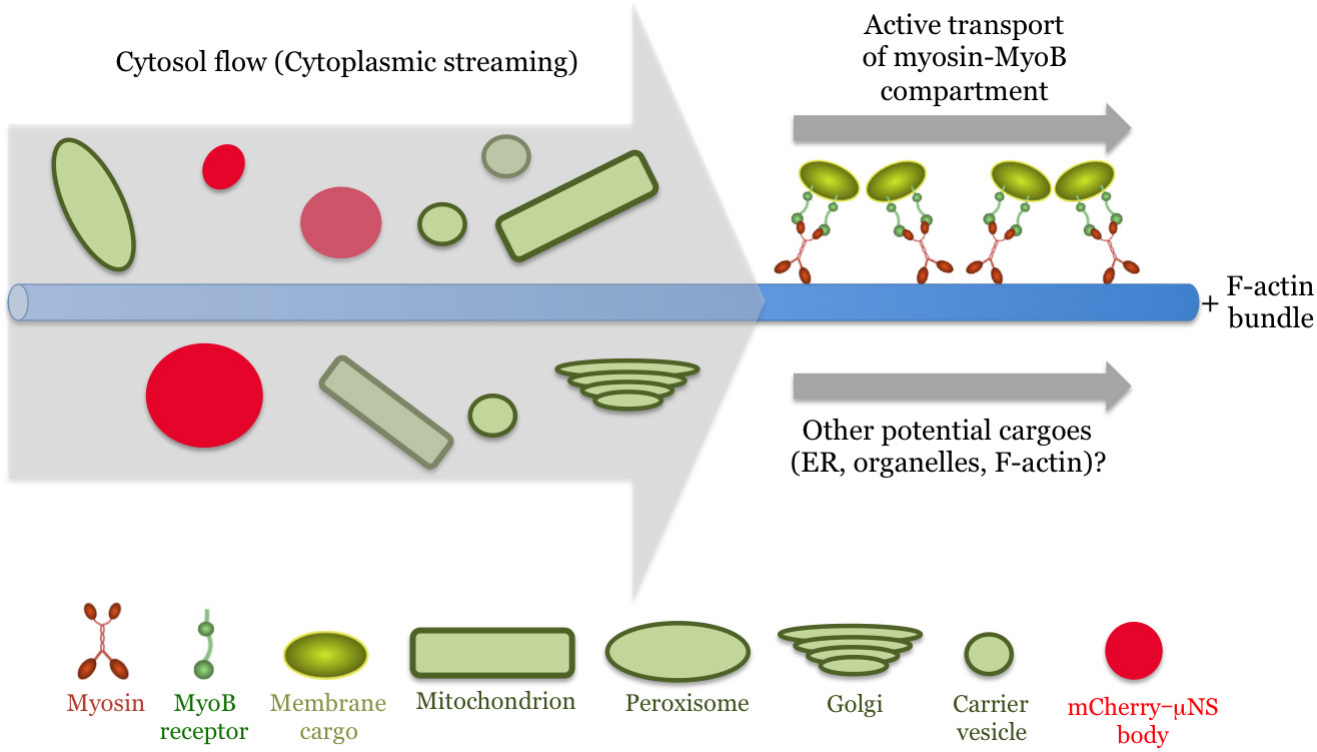
Model of two major particle trafficking mechanisms in plant cells. The active, myosin-driven transport of the MyoB membrane compartment along the F-actin bundles entrains cytosolic flow, which carries various organelles, vesicles and inert tracer mCherry-**μ**NS bodies.

Conspicuously, overexpression of both the myosin XI-K cargo-binding domain and the myosin-binding DUF593 domains of MyoB1 or MyoB2 resulted in the inhibition of the transport of not only the MyoB1 compartment, but also of each of the other five investigated cargoes. The extents of velocity reduction were very similar for all six cargoes, and characteristic of the particular overexpressed inhibitor, not the specific cargo (Fig 1). Because both inhibition approaches target the myosin-MyoB interactions, this outcome implies that the MyoB compartment is a major driver responsible for movements of each of the other five cargoes including the mCherry-**μ**NS bodies (Fig 7).

An alternative interpretation evoking competition of the dominant negative inhibition effector (DUF593 in our case) with myosin binding of four distinct organellar receptors analogous to that observed for some yeast myosin V cargoes [39] does not explain inhibition of the receptor-less **μ**NS-mCherry trafficking. This interpretation is also incompatible with the reduction of the streaming velocity in a quadruple MyoB1-4 knockout mutant because elimination of MyoB receptors cannot affect potential myosin interactions with other cargoes. (S2 Fig). Yet additional support for the model shown in Fig 7 is provided by localization of the myosin XI-K [29], MyoB1, MyoB2 and MyoB7 to similar vesicle-like compartments that are distinct from larger organelles [30]. Thus, it can be concluded that myosins XI cooperate with MyoB receptors to provide the driving force and the cargo to launch vigorous cytoplasmic streaming in plant cells. This conclusion sheds light on the molecular mechanism of a process that has been studied for decades but so far eluded a firm mechanistic explanation, as well as focuses future research on investigation of the MyoB compartment and other potential myosin cargoes that contribute to cytoplasmic streaming.

How does this outcome explains the saltatory trafficking mode of organelles such as Golgi [31,32]? It was broadly assumed that this mode depended on the transient associations between organelles and myosin motors, an assumption we shared in our previous interpretation of streaming as localized cytosol intermixing by ‘hopping’ organelles [22]. Identification of the MyoB compartment as the principal myosin cargo challenged this assumption and implied that the myosin-MyoB compartment moves actively along F-actin exerting cytoplasmic streaming that carries other membrane cargoes [30,33]. These passively transported cargoes would, by necessity, travel only when they enter the cytoplasmic streams leaving large fraction of the cargoes nearly immotile (Fig 2). Therefore, based on the genetic and particle trafficking analyses presented in this work, we revised our previous interpretation of streaming [22] that is now modeled as a network of streams flowing along the F-actin bundles (Fig 7).

It should be mentioned that here we investigate the transport of several particulate membrane compartments, but not of the reticulate ER network, which warrants a separate study. Previous work has shown that the ER flow depends on myosins XI and that the myosins contribute to ER organization [26,28,40]. Notably, a cytoskeleton-independent ER remodeling process involving RHD3 is capable of modulating both the ER flow and organelle velocities [35]. These data, however, does not allow distinguishing between two possible mechanisms of ER transport: i) passive flow with the cytoplasmic streaming with RHD3 modulating the hydrodynamic properties of the ER; ii) active transport mediated by myosins and yet unknown ER-specific receptors. Possible existence of such receptors is suggested by association of FLOURY 1 (a member of the MyoB family) with the specialized ER subdomain surrounding the protein bodies in the maize kernels [41]. However, no data implicating this protein in ER mobilization exist so far, whereas MyoB1, MyoB2, and MyoB7 receptors expressed throughout Arabidopsis plants, as well as pollen tube-specific MyoB from *N*. *tabacum* [42], do not localize to the ER. Thus, the very existence of ER-specific myosin receptors capable of driving ER flow remains an open question. Furthermore, as concluded in computer simulation models [7,43] and a following study [44], movement of the small cargoes by myosins exerts sufficient drag on cytosol to establish cytoplasmic streaming with no need in additional coupling of myosins to ER network.

Recently, the nuclear, myosin XI-I-specific, receptors WIT1/2 unrelated to MyoBs were implicated in a slow cyclical nuclear repositioning [45]. This process, however, is unlikely to contribute to fast and perpetual cytoplasmic streaming. Another potential myosin receptor is a processing body-associated protein DCP1, but the role of processing bodies in cytoplasmic streaming is yet to be investigated [46]. Thus, although the evidence for the drivers of streaming other than MyoBs is lacking, potential contributions from such drivers are not excluded with existing data (Fig 7).

Another important conclusion of this work is functional link between the MyoB-dependent cytoplasmic streaming and cell expansion, plant growth and morphogenesis. Using synthetic quintuple gene knockouts in which two myosin and three *MyoB* genes were inactivated, we demonstrate that the genetic interactions between myosins and their cognate MyoB receptors contribute to the expansion of two distinct cell types (Fig 3). These effects phenocopy triple and quadruple myosin knockouts [28], showing that myosin functions in cell expansion are executed in cooperation with MyoB receptors. Interestingly, such synergistic action of myosins and MyoBs was not detected for root hairs that grow in a polarized manner (S3 Fig). This outcome could be explained by the involvement of distinct MyoB subsets in polarized cell growth, a possibility supported by the root hair-specific expression of MyoB family members AT1G74830 and AT5G57830 [47]. In general, because individual myosins XI and MyoBs both exhibit tissue-specific expression patterns [30,37], particular configurations of myosin-MyoB complexes could generate cytoplasmic streaming patterns optimal for different cell types.

The reduced cell expansion in s5KOs also resulted in defective leaf and stem growth (Fig 4), abnormal stem morphology (Fig 5A and 5B), and delayed transition from growth to flowering (Fig 6) making the myosin-MyoB compartment an important determinant of the cell and organism size, as well as a major contributor to plant development.

As demonstrated earlier, the myosin-MyoB compartment does not correspond to Golgi, other larger organelles, or to major vesicular compartments, including trans-Golgi network, secretory vesicles, endocytic vesicles, or the pre-vacuolar compartment [29,30]. Treatment with brefeldin A that disrupts the ER-to-Golgi-to-plasma membrane biosynthetic pathway [48,49] does not affect MyoB compartment [30]. Taken together with the results described here, these data strongly support a specialized transport function for the myosin-MyoB compartment that acts as a major driver of cytoplasmic streaming. This, in turn, implies that the main role of streaming in plants is to speed up the delivery of organelles and carrier vesicles and to elevate the metabolic status of the cell by cytosol circulation. At the cellular and organismal levels, this role translates into contributions to cell expansion and to plant growth, as defined by the mutant phenotypes observed. It should be noted that a comprehensive investigation of the protein and lipid composition of the MyoB compartment is needed to understand its biogenesis and relationship to other membrane compartments.

Because the MyoB receptors are universally conserved in land plants [30], it seems likely that the emergence of the MyoB family concomitant with proliferation of myosins XI have preconditioned vigorous cytoplasmic streaming that is one of the most prominent features of plant cells. In turn, acceleration of cell growth due to streaming [18] helped enable the development of larger plants. This evolutionary line of causation is further strengthened by the presence of a MyoB receptor homolog in the filamentous green alga *Nitella mirabilis* (accession No JV762366.1, NCBI Transcriptome Shotgun Assembly sequences database), one of the *Charales*, an order of algae in which both the fastest known myosins [19] and the fastest cytoplasmic streaming [20] have been characterized. These are within the *Charophyta*, an algal lineage most closely related to land plants [50]. Intriguingly, sequenced genomes within the deeper branching *Chlorophyta* algae (e.g., *Chlamydomonas reinhardtii*) lack MyoB homologs suggesting that this myosin receptor appeared as an evolutionary innovation in the direct algal ancestors of land plants.

In conclusion, here we identify one of the major mechanisms of intracellular dynamics in plants. We demonstrate that cytoplasmic streaming is a primary route for organelle and carrier vesicle transport. We further show that cytoplasm is entrained by a distinct membrane compartment transported by direct linkage of the MyoB membrane-anchored receptors to myosins XI (Fig 7). Finally, we reveal that the functional cooperation between myosins and MyoB receptors contributes to cell expansion, plant growth and morphogenesis. Because organelle trafficking and cytoplasmic streaming occur both in growing and fully differentiated cells, these processes also facilitate cell homeostasis. Thus, this molecular motor- and membrane receptor-dependent transport emerges as a critical aspect of plant biology.

## Supporting Information

S1 Fig Transient expression and subcellular localization of MyoB1-GFP, MyoB2-GFP and mCherry-μNS in *Nicotiana benthamiana*. (A) Confocal laser scanning microscopy (CLSM) images of the membrane compartments tagged by MyoB1-GFP and MyoB2-GFP of *N*. *benthamiana* 2 days post agrobacterial infiltration. Note similar localization patterns for both compartments present mostly in linear arrays of small bodies similar to their Arabidopsis orthologs described earlier (Peremyslov et al., 2013). (B) Top panel: Immunoblot analysis of the ectopically expressed, 3 x hemagglutinin (HA) epitope-tagged proteins using rabbit polyclonal HA-specific antibody (**α**-HA Ab). DUF593 MyoB1 and MyoB2, fragments of *N*. *benthamiana* MyoB1 and MyoB2 including myosin-binding DUF593 domains of MyoB1 and MyoB2 and following protein regions up to corresponding C-termini; GUS, bacterial **β**-glucuronidase (control); XI-K GTD, coiled coil and cargo-binding globular tail domains (GTD) of *N*. *benthamiana* myosin XI-K. Bottom panel: Actin levels in the samples from the bottom part of immunoblot shown above detected using mouse actin-specific rabbit polyclonal antibody used as protein loading controls. (C) CLSM image of the variable size cytoplasmic inclusion bodies formed upon ectopic expression of the inert tracer protein mCherry-**μ**NS_C_. Scale bar, 5 **μ**m. (D) Pull-down assays showing specific binding of MyoB1 DUF593 domain (fused with GFP; DUF593-GFP) to immobilized myosin XI-K GTD fused with maltose-binding protein (MBP-XIK GTD), but not to immobilized MBP-GUS control. Note that GFP- **μ**NS_C_ does not bind to either MBP-GUS or to MBP-XIK GTD. The rabbit, polyclonal, GFP-specific antibody (**α**-GFP Ab) was used to detect each GFP-fused protein before (Input) and after pull down assays with the resin-immobilized MBP-GUS or MBP-XIK GTD.
(TIFF)

S2 Fig Mean velocity of mitochondria in the epidermal cells of three Arabidopsis lines: Columbia, quadruple knockout mutant *myob1 myob2 myob3 myob4* and triple knockout mutant *xi-k xi-1 xi-2*. The number of individual mitochondria velocity of which was measured in each plant line was 500. Two and three asterisks denote statistical significance of p<0.05 and p<0.001, respectively. Large standard deviations are due to great variability in velocities of individual organelles many of which are nearly immotile whereas others move at high velocities.
(TIFF)

S3 Fig Mean length and standard deviation of root hairs after 5 days of growth on vertical agar plates containing MS medium and 2% sucrose (n = 325 root hairs from 16 roots for each variant). The genotype for each of the six experimental variants is shown below the columns (*myob1-3* corresponds to triple gene knockout *myob1 myob2 myob3*). Note that although elimination of the three MyoB genes does result in a modest, 9% reduction in root hair length, there is no synergistic effects due to elimination of these genes in *xi-k xi-1* or *xi-k xi-2* backgrouns, plausibly due to the dominant effect of myosin gene elimination.
(TIFF)

S4 Fig Simultaneous elimination of two myosins and three MyoB receptors in synthetic quintuple mutants has synergistic effect on primary root morphology but not on a growth rate. (A) Primary root growth rate (mm added in 24 hrs) in six distinct genetic backgrounds as indicated at the bottom. Note an absence of statistically significant difference in growth rates between each of the quintuple synthetic mutant and corresponding controls (triple *myob* mutant and double myosin mutants). Error bars are standard deviations. n = 30 for each variant. (B) To quantify the wavy phenotype of root growth, the length of root added from day 5 to day 7 was divided by linear length the root tip had been displaced during this time. This ratio (an Index of Waviness) will have a value of 1 if root growth is strictly linear, but will be greater than one to the degree that the growth was wavy. The ratio incorporates the results of both an increased frequency or amplitude of waviness. This parameter was evaluated for each of the genetic backround as indicated. Error bars represent standard deviation. The combination of the *myob1-3* mutations with *xi-k xi-1* or *xi-k xi-2* double myosin mutants in synthetic quintuple mutants led to significantly increased waviness compared to either the *myob1-3* or the double myosin mutations alone (p<10^−5^, one-sided t-test, n = 30). (C) Images of 7 days-old seedlings showing significant increase in waviness due to synergistic effect of MyoB1-3 and two myosins elimination in synthetic quintuple mutants *xi-k xi-1 myob1 myob2 myob3* or *xi-k xi-2 myob1 myob2 myob3*.
(TIFF)

S5 Fig Rescue of growth defects in the synthetic quadruple knockout mutant *xi-k myob1-3* by stably expressing MyoB1:YFP or MyoB2:YFP. (A) Representative images of the aerial parts of control and mutant plants; note abnormal stem morphology in *xi-k myob1-3* versus the control Columbia-0 and each of the rescue variants. (B) Mean plant heights and standard deviations measured at 52 days post germination.
(TIFF)

S1 Movie Trafficking of the *N*. *benthamiana* MyoB1-GFP-tagged endomembrane compartment under conditions of dominant negative inhibition. (A) Co-expression of MyoB1-GFP and **β**-glucuronidase (GUS; a control for dominant negative inhibition). (B) Co-expression of MyoB1-GFP and myosin XI-K globular tail domain (GTD). (C) Co-expression of MyoB1-GFP and MyoB1 DUF593, the myosin-binding domain of MyoB1. (D) Co-expression of MyoB1-GFP and MyoB2 DUF593, the myosin-binding domain of MyoB2. Each movie represents 20 frames at 0.47 sec per frame. Image dimensions are x: 41.5 **μ**m; y: 41.5 **μ**m.
(MP4)

S2 Movie Trafficking of the Golgi stacks under conditions of dominant negative inhibition. (A) Co-expression of the GFP-tagged Golgi-specific marker NAG-GFP (the transmembrane stem region of N-acetylglucosaminyl transferase I) and GUS. (B) Co-expression of NAG-GFP and myosin XI-K globular tail domain (GTD). (C) Co-expression of NAG-GFP and MyoB1 DUF593. (D) Co-expression of NAG-GFP and MyoB2 DUF593. Each movie represents 20 frames at 0.94 sec per frame. Image dimensions are x: 41.5 **μ**m; y: 41.5 **μ**m.
(MP4)

S3 Movie Trafficking of the mCherry-μNS protein inclusion bodies under conditions of dominant negative inhibition. (A) Co-expression of the mCherry- **μ**NS_C_ and GUS. (B) Co-expression of the mCherry-**μ**NS_C_ and myosin XI-K globular tail domain (GTD). (C) Co-expression of the mCherry-**μ**NS_C_ and MyoB1 DUF595. (D) Co-expression of the mCherry-**μ**NS_C_ and MyoB2 DUF593. Each movie represents 20 frames at 0.56 sec per frame. Image dimensions are x: 41.5 **μ**m; y: 41.5 **μ**m.
(MP4)
