# Supplementary figures and images for: Myosin-Powered Membrane Compartment Drives Cytoplasmic Streaming, Cell Expansion and Plant Development
Source: PLoS One. 2015 Oct 1;10(10):e0139331. doi: 10.1371/journal.pone.0139331 (PMC4591342; doi:10.1371/journal.pone.0139331)

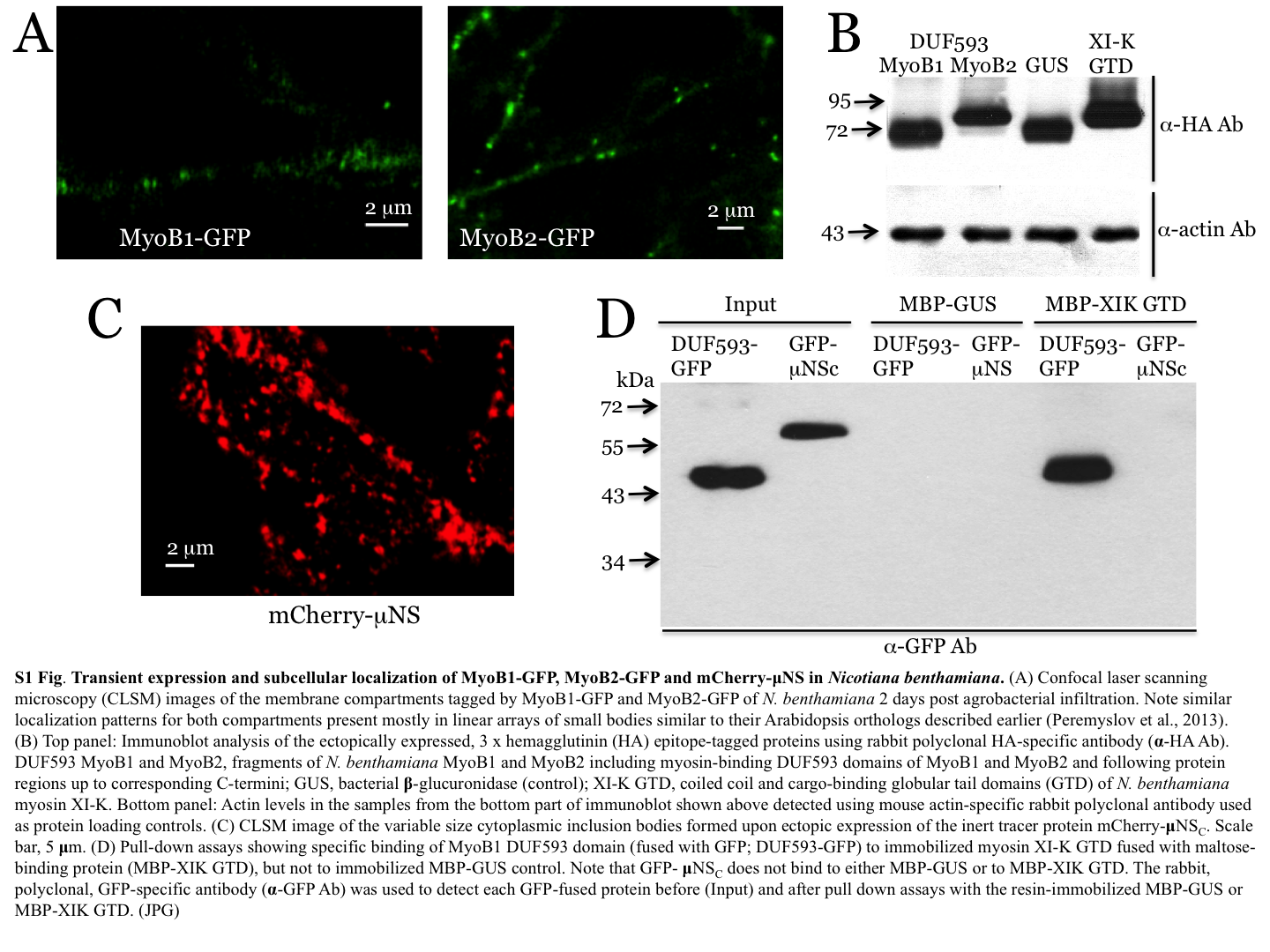

Supplement: S1 Fig — (A) Confocal laser scanning microscopy (CLSM) images of the membrane compartments tagged by MyoB1-GFP and MyoB2-GFP of N. benthamiana 2 days post agrobacterial infiltration. Note similar localization patterns for both compartments present mostly in linear arrays of small bodies similar to their Arabidopsis orthologs described earlier (Peremyslov et al., 2013). (B) Top panel: Immunoblot analysis of the ectopically expressed, 3 x hemagglutinin (HA) epitope-tagged proteins using rabbit polyclonal HA-specific antibody (α-HA Ab). DUF593 MyoB1 and MyoB2, fragments of N. benthamiana MyoB1 and MyoB2 including myosin-binding DUF593 domains of MyoB1 and MyoB2 and following protein regions up to corresponding C-termini; GUS, bacterial β-glucuronidase (control); XI-K GTD, coiled coil and cargo-binding globular tail domains (GTD) of N. benthamiana myosin XI-K. Bottom panel: Actin levels in the samples from the bottom part of immunoblot shown above detected using mouse actin-specific rabbit polyclonal antibody used as protein loading controls. (C) CLSM image of the variable size cytoplasmic inclusion bodies formed upon ectopic expression of the inert tracer protein mCherry-μNSC. Scale bar, 5 μm. (D) Pull-down assays showing specific binding of MyoB1 DUF593 domain (fused with GFP; DUF593-GFP) to immobilized myosin XI-K GTD fused with maltose-binding protein (MBP-XIK GTD), but not to immobilized MBP-GUS control. Note that GFP- μNSC does not bind to either MBP-GUS or to MBP-XIK GTD. The rabbit, polyclonal, GFP-specific antibody (α-GFP Ab) was used to detect each GFP-fused protein before (Input) and after pull down assays with the resin-immobilized MBP-GUS or MBP-XIK GTD. (TIFF) [file pone.0139331.s001.tiff]

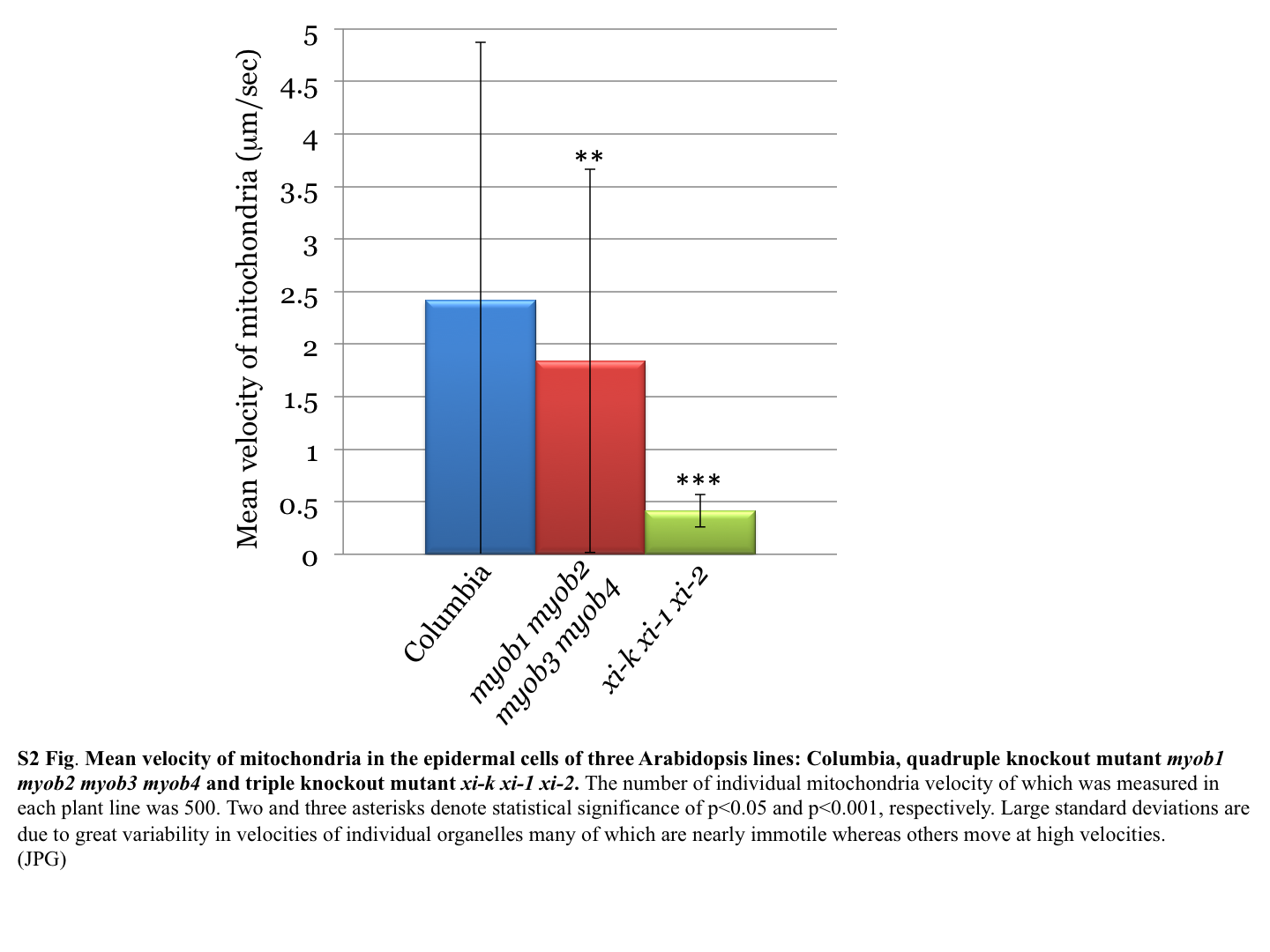

Supplement: S2 Fig — The number of individual mitochondria velocity of which was measured in each plant line was 500. Two and three asterisks denote statistical significance of p<0.05 and p<0.001, respectively. Large standard deviations are due to great variability in velocities of individual organelles many of which are nearly immotile whereas others move at high velocities. (TIFF) [file pone.0139331.s002.tiff]

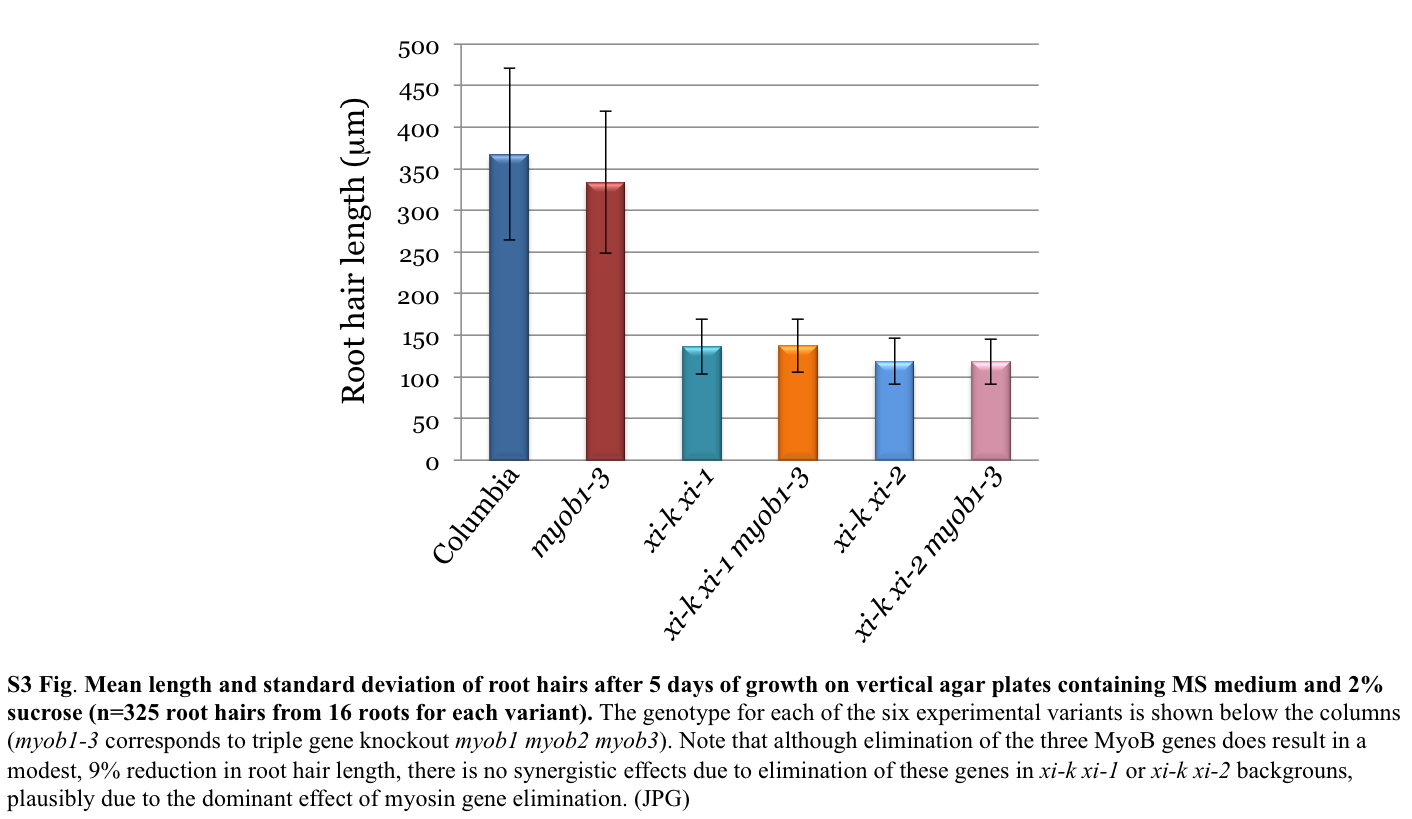

Supplement: S3 Fig — The genotype for each of the six experimental variants is shown below the columns (myob1-3 corresponds to triple gene knockout myob1 myob2 myob3). Note that although elimination of the three MyoB genes does result in a modest, 9% reduction in root hair length, there is no synergistic effects due to elimination of these genes in xi-k xi-1 or xi-k xi-2 backgrouns, plausibly due to the dominant effect of myosin gene elimination. (TIFF) [file pone.0139331.s003.tiff]

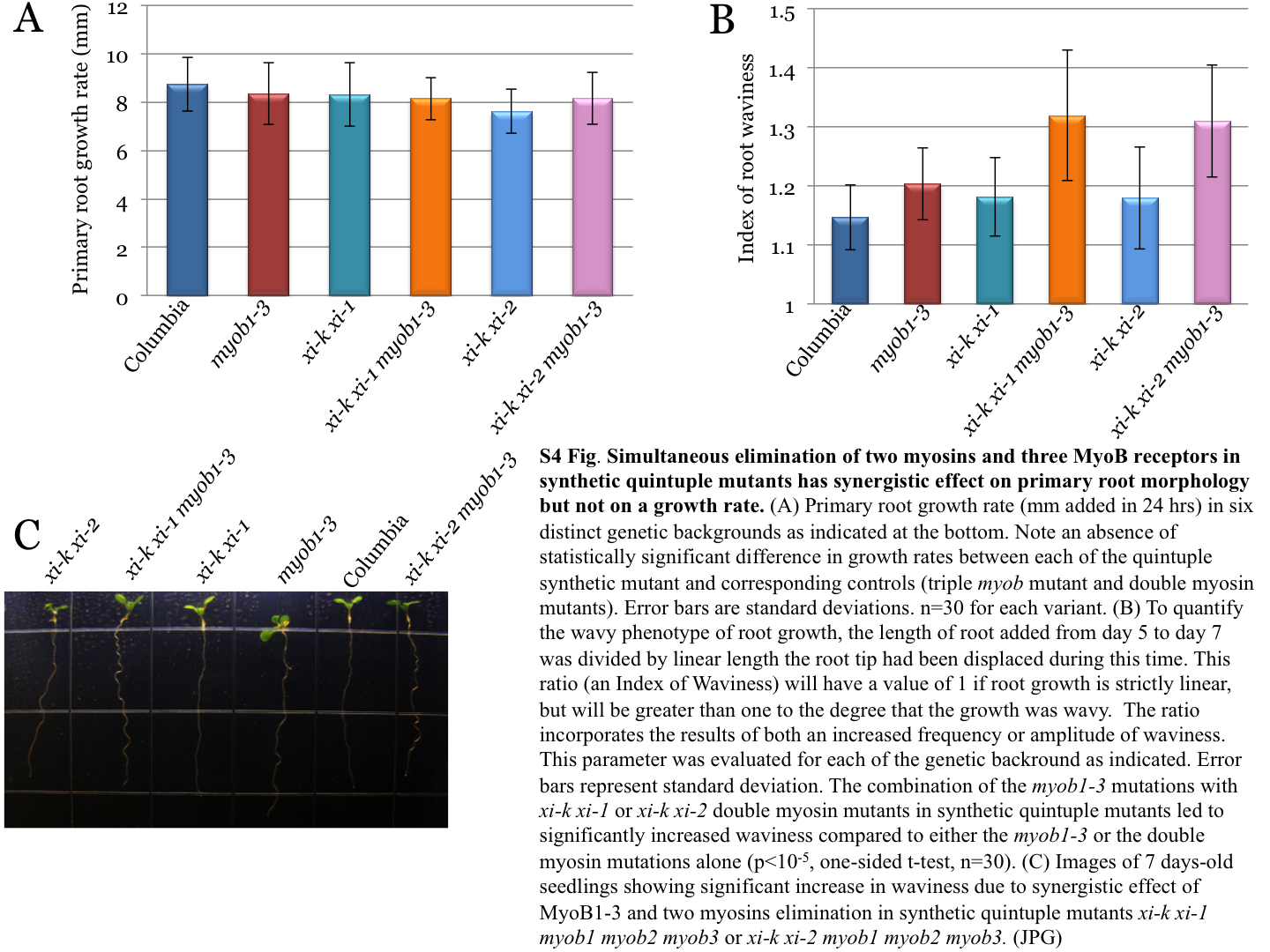

Supplement: S4 Fig — (A) Primary root growth rate (mm added in 24 hrs) in six distinct genetic backgrounds as indicated at the bottom. Note an absence of statistically significant difference in growth rates between each of the quintuple synthetic mutant and corresponding controls (triple myob mutant and double myosin mutants). Error bars are standard deviations. n = 30 for each variant. (B) To quantify the wavy phenotype of root growth, the length of root added from day 5 to day 7 was divided by linear length the root tip had been displaced during this time. This ratio (an Index of Waviness) will have a value of 1 if root growth is strictly linear, but will be greater than one to the degree that the growth was wavy. The ratio incorporates the results of both an increased frequency or amplitude of waviness. This parameter was evaluated for each of the genetic backround as indicated. Error bars represent standard deviation. The combination of the myob1-3 mutations with xi-k xi-1 or xi-k xi-2 double myosin mutants in synthetic quintuple mutants led to significantly increased waviness compared to either the myob1-3 or the double myosin mutations alone (p<10−5, one-sided t-test, n = 30). (C) Images of 7 days-old seedlings showing significant increase in waviness due to synergistic effect of MyoB1-3 and two myosins elimination in synthetic quintuple mutants xi-k xi-1 myob1 myob2 myob3 or xi-k xi-2 myob1 myob2 myob3. (TIFF) [file pone.0139331.s004.tiff]

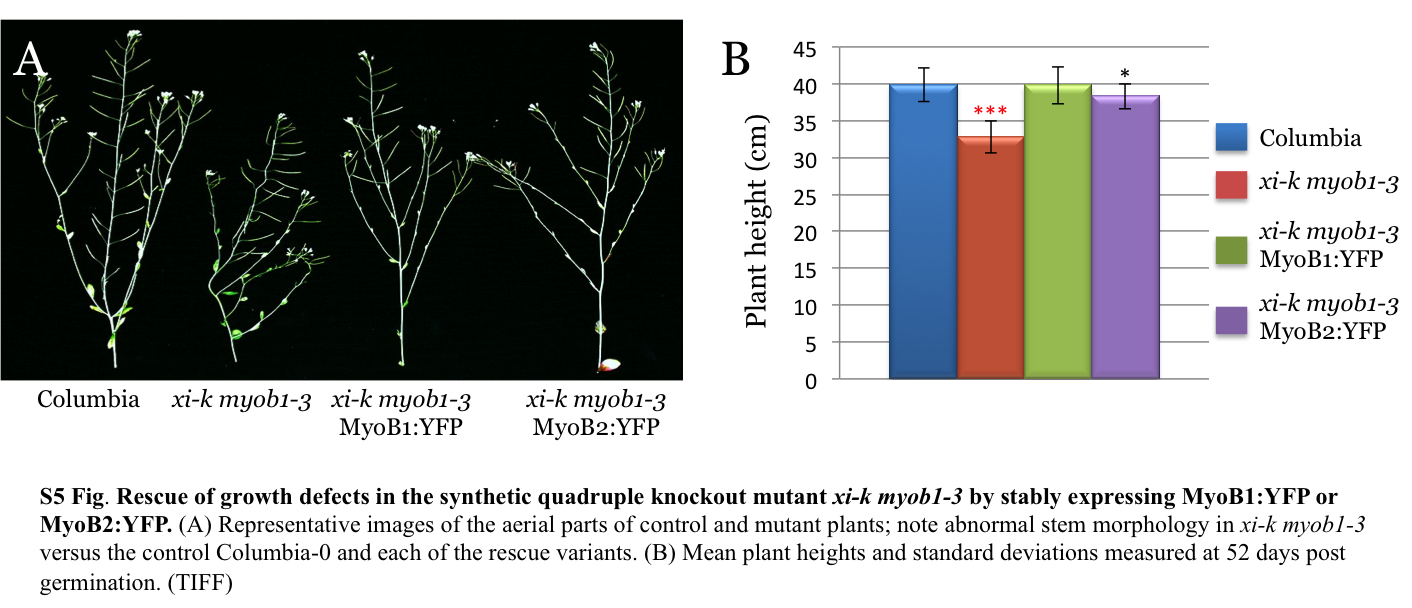

Supplement: S5 Fig — (A) Representative images of the aerial parts of control and mutant plants; note abnormal stem morphology in xi-k myob1-3 versus the control Columbia-0 and each of the rescue variants. (B) Mean plant heights and standard deviations measured at 52 days post germination. (TIFF) [file pone.0139331.s005.tiff]
